# Does autoimmune diseases increase the risk of frailty? A Mendelian randomization study

**DOI:** 10.3389/fendo.2024.1364368

**Published:** 2024-08-27

**Authors:** Jinlei Zhou, Yuan Zhang, Ting Ni, Yanlei Li, Haiyu Shao, Fei Wang, Sen Xu, Yazeng Huang, Jun Zhang, Tingxiao Zhao

**Affiliations:** ^1^ Second Clinical Medical College, Zhejiang Chinese Medical University, Hangzhou, Zhejiang, China; ^2^ Center for Plastic & Reconstructive Surgery, Department of Orthopedics, Zhejiang Provincial People’s Hospital (Affiliated People’s Hospital, Hangzhou Medical College), Hangzhou, Zhejiang, China; ^3^ Department of Rheumatology and Immunology, The Second Affiliated Hospital of Zhejiang Chinese Medical University, Second Clinical Medical College, Zhejiang Chinese Medical University, Hangzhou, Zhejiang, China; ^4^ The First Affiliated Hospital of Soochow University, Suzhou, Jiangsu, China; ^5^ Clinical Medical College, Hangzhou Medical College, Hangzhou, Zhejiang, China; ^6^ Department of Orthopedics, Zhejiang Provincial People’s Hospital Bijie Hospital, Bijie, Guizhou, China

**Keywords:** autoimmune disease, frailty, Mendelian randomization, hypothyroidism, hyperthyroidism, rheumatoid arthritis, type 1 diabetes, multiple sclerosis

## Abstract

**Background:**

The causality of autoimmune diseases with frailty has not been firmly established. We conducted this Mendelian randomization (MR) study to unveil the causal associations between autoimmune diseases with frailty.

**Methods:**

A MR analyses were performed to explore the relationships between autoimmune disease and frailty, using summary genome-wide association statistics.

**Results:**

Through a comprehensive and meticulous screening process, we incorporated 46, 7, 12, 20, 5, and 53 single nucleotide polymorphisms (SNPs) as instrumental variables (IVs) for hypothyroidism, hyperthyroidism, rheumatoid arthritis (RA), type 1 diabetes (T1D), multiple sclerosis (MS), and overall autoimmune disease, respectively. Our analysis revealed that hypothyroidism (OR = 1.023, 95% CI: 1.008–1.038, p = 0.0015), hyperthyroidism (OR = 1.024, 95% CI: 1.004–1.045, p = 0.0163), RA (OR = 1.031, 95% CI: 1.011–1.052, p = 0.0017), T1D (OR = 1.011, 95% CI: 1.004–1.017, p = 0.0012), and overall autoimmune disease (OR = 1.044, 95% CI: 1.028–1.061, p = 5.32*10^-8) exhibited a positive causal effect on frailty. Conversely, there may be a negative causal association between MS (OR = 0.984, 95% CI: 0.977–0.992, p = 4.87*10^-5) and frailty. Cochran’s Q test indicated heterogeneity among IVs derived from hypothyroidism, hyperthyroidism, T1D, and overall autoimmune diseases. The MR-Egger regression analyzes revealed an absence of horizontal pleiotropy in any of the conducted analyses.

**Conclusion:**

This study elucidates that hypothyroidism, hyperthyroidism, RA, T1D, and overall autoimmune disease were linked to an elevated risk of frailty. Conversely, MS appears to be associated with a potential decrease in the risk of frailty.

## Introduction

Frailty manifests as a clinicopathological condition intricately linked to the depletion of biological reserves across diverse organ systems, concomitant with the dysregulation of homeostatic mechanisms (1). This condition increases the risk of adverse outcomes, including multimorbidity, disability, and mortality, and places a substantial economic strain on social healthcare system. Evidence suggests that approximately 10% of individuals aged > 65 years manifest frailty, while among those aged > 85 years, frailty is observed in a range extending from 25% to 50% of the population (2). Although frailty is common in older individuals, it is not an inevitable outcome of aging; instead, it can be regarded as a pathological condition of rapid aging (3). Hence, health professionals must account for the potential of frailty in the diagnosis and treatment of illnesses, to accelerate patient recuperation and alleviate the financial burden of healthcare.

**Figure 1:**
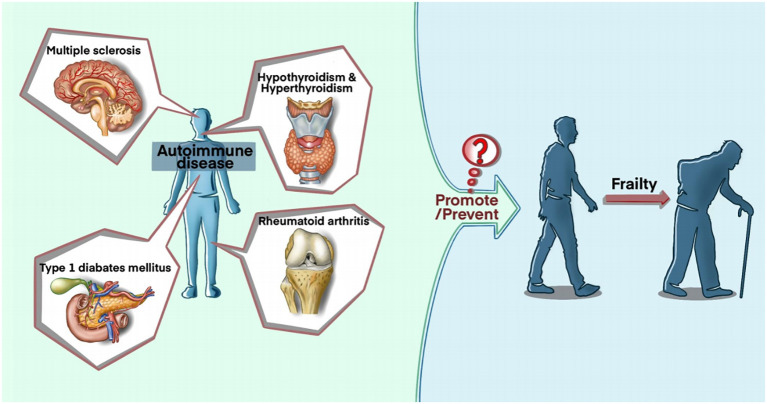
A General Schematic on whether autoimmune diseases contribute to the development of frailty.

Autoimmune diseases are characterized by the generation of antibodies that specifically target host tissues or immune effector cells displaying autoreactivity toward endogenous peptides. There are many inflammatory mediators released during the autoimmune response, including inflammatory cytokines such as interleukin-6 (IL-6) and tumor necrosis factor (TNF-1), which hasten the onset of frailty (4). Furthermore, numerous epidemiological studies demonstrate a significant association between frailty and various autoimmune diseases, such as hypothyroidism, hyperthyroidism, rheumatoid arthritis (RA), type 1 diabetes (T1D), and multiple sclerosis (MS) (5–9). However, observational studies are susceptible to the complexities of reverse causation and a plethora of confounding variables, including factors such as depression, smoking, and physical function (9, 10). Consequently, further research is necessary to determine whether autoimmune diseases contribute to the development of frailty (**Figure 1**).

Mendelian randomization (MR) is a methodological approach employing genetic variants as instrumental variables (IVs) to interrogate the congruence of an observational correlation between an exposure and an outcome with a potential causal inference. This approach minimizes the impact of confounding variables and avoids the potential distortion arising from reverse causation bias. Using a two-sample MR analysis, our study assessed the causal relationship between frailty and overall autoimmune disease, as well as five commonly occurring autoimmune diseases (hypothyroidism, hyperthyroidism, RA, T1D, and MS).

## Methods

### Research design

**Figure 2:**
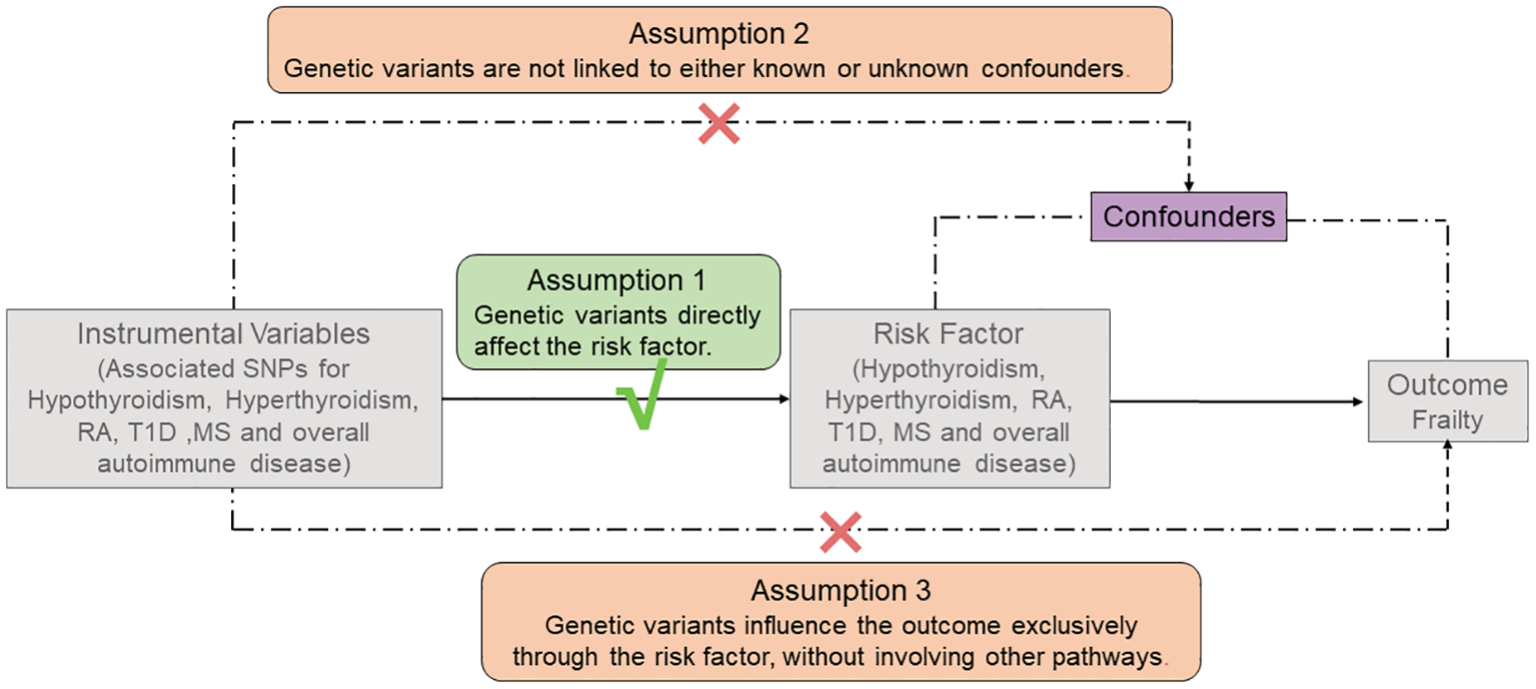
The three main assumptions of Mendelian Randomization analysis. RA, rheumatoid arthritis; T1D, type 1 diabetes; MS, Multiple sclerosis.

Three key assumptions must be satisfied to conduct an MR study, as shown in **Figure 2**. First, the screened genetic variants, available as potential IVs, must exhibit a robust correlation with exposure. Second, the screened genetic variants cannot be associated with confounders. Third, the genetic variants exclusively influence outcomes through exposure, devoid of alternative pathways (11, 12).

### Database sources for autoimmune diseases (exposure)

Through an exhaustive exploration within the FinnGen R9 database, we obtained six datasets associated with autoimmune diseases. Hence, no additional ethical authorizations were deemed necessary. In addition, we confined the genetic makeup of the study cohort solely to individuals of European ancestry, thus mitigating any bias that may have arisen from interpopulation blending.

The dataset relating to hypothyroidism (GAWS ID: finn-b-E4_HYTHY_AI_STRICT) consists of 198,472 individuals of European origin, including 22,997 cases and 175,475 healthy controls; the dataset related to autoimmune hyperthyroidism (GAWS ID: finn-b-AUTOIMMUNE_HYPERTHYROIDISM) comprises a sample size of 173,938 individuals, among which there were 962 cases and 172,976 healthy controls; the dataset for RA (GAWS ID: finn-b-RHEUMA_SEROPOS_WIDE) comprises 218,790 samples, with 4,594 cases and 214,196 controls; the dataset for T1D (GAWS ID: finn-b-T1D_WIDE1) comprises 185,571 samples, consisting of 4,849 cases and 180,722 controls; the dataset for MS (GAWS ID: finn-b-G6_MS) comprises 218,189 samples, which encompasses 1,048 cases and 217,141 controls.

Similarly, the FinnGen R9 database provides the overall autoimmune disease dataset (GAWS ID: finn-b-AUTOIMMUNE), with a total sample size of 218,792 participants, comprising 42,202 cases and 176,590 controls. The dataset comprises 45 autoimmune diseases, with further information provided in **Supplementary Table S1**.

### Database sources for frailty (outcome)

The frailty data was obtained from a publicly available GWAS dataset (GWAS ID: ebi-a-GCST90020053), consisting of a cohort of 175,226 individuals of European ancestry. A GWAS meta-analysis of the Frailty Index among UK Biobank participants (n = 164,610, aged 60–70 years) and Swedish TwinGene participants (n = 10,616, aged 41–87 years) was conducted by Janice et al (13). The sources and relevant details for the exposure and outcome samples can be found in **Supplementary Table S2**.

### Selection of instrumental variables

**Figure 3:**
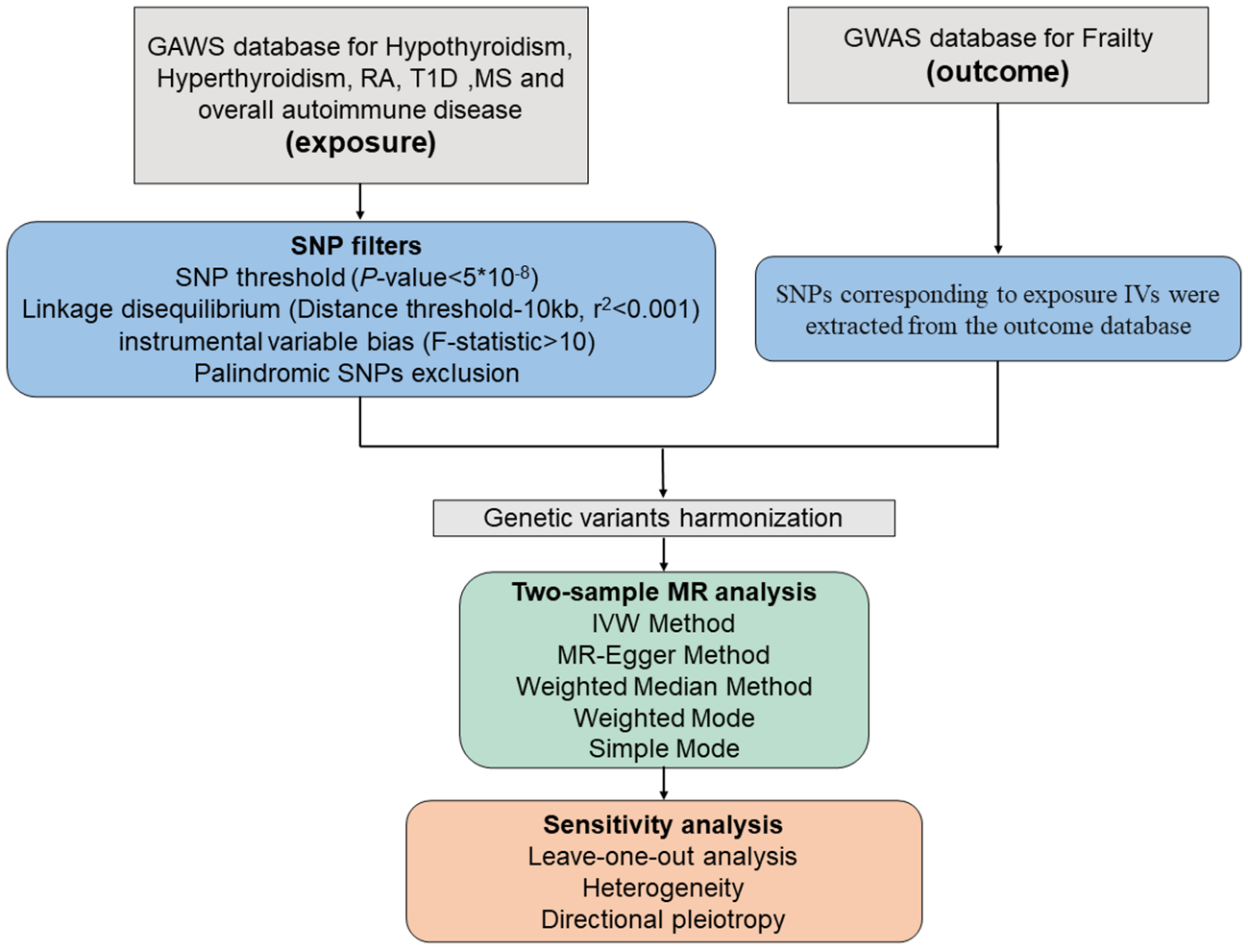
Schematic diagram of the Mendelian Randomization analysis process. RA, rheumatoid arthritis; T1D, type 1 diabetes; MS, Multiple sclerosis.

Several screening procedures for single nucleotide polymorphisms (SNPs) were conducted to ensure the reliability of our findings regarding the causal link between autoimmune disorders and frailty, as detailed in the process in **Figure 3**. Firstly, only SNPs meeting the rigorous threshold of genome-wide significance (p < 5*10^-8) were used as IVs, out of the six GWAS datasets associated with autoimmune diseases. Secondly, the PLINK clumping method was used to preserve independent SNPs (r^2 < 0.001, kb = 10,000) (14). Thirdly, the strength of the IVs was assessed by calculating the F-statistic for each SNP. An F-statistic > 10 indicates the absence of vulnerability to weak IV bias, signifying a strong correlation between IVs and exposure (15, 16). Fourthly, we excluded SNPs that were absent from the outcome dataset or displayed allele inconsistencies between exposure and outcome. Fifthly, SNPs displaying palindromic characteristics were removed. Sixthly, we conducted a search on the PhenoScanner website (http://www.phenoscanner.medschl.cam.ac.uk/) for all relevant SNP phenotypes and excluded SNPs related to confounding factors (depression, irritable bowel syndrome, type 2 diabetes, physical function, disease activity, Hodgkin’s lymphoma, coronary artery disease, smoking, and bone mineral density) and other autoimmune diseases (p < 5*10^-8) (9, 10).

### Statistical analysis

The *“TwoSampleMR”* software (version 0.5.7) from the R program (version 4.2.2) was used to examine the hypothetical causal association between autoimmune diseases and frailty. For establishing the causal association between autoimmune diseases and frailty, the inverse variance weighted (IVW) method was used as the primary analysis (17). Moreover, our MR analysis integrated the MR-Egger regression, Weighted Median Estimator (WME), Weighted Mode method, and Simple Mode method, thus ensuring a comprehensive and robust assessment of the causal linkage (11, 15, 17, 18).

Furthermore, Cochran’s Q test was conducted employing both the IVW and MR Egger methods to assess statistical heterogeneity among the SNPs. The random-effects IVW was employed if heterogeneity was statistically significant (p < 0.05); the fixed-effects IVW was used if it was not (19). Horizontal pleiotropy manifests when a genetic variation exhibits associations with diverse phenotypes through distinct pathways, potentially compromising the reliability of MR analysis (20, 21). Therefore, we employed MR-Egger regressions to identify and disclose probable confounders that could potentially skew the MR horizontal pleiotropy manifests when a genetic variation exhibits associations with diverse phenotypes through distinct pathways, potentially compromising the reliability of MR analysis. We also performed a leave-one-out analysis to determine if any specific independent variable contributed significantly to the causal link between exposure and outcome. In addition, we utilized MR-PRESSO to identify any outliers and examined the discrepancies in outcomes before and after removal of outliers (18). Furthermore, we calculated the F-statistics (formula: R^2^=(2*EAF*(1-EAF)*beta^2^)/[(2*EAF*(1-EAF)*beta^2^)+(2*EAF*(1-EAF)*N*SE)]; F=R^2^*(N-2)/(1-R^2^)) of the SNPs (22). Finally, we conducted a reverse MR analysis, wherein frailty served as the exposure and the six autoimmune conditions as outcomes. This approach was instrumental in mitigating the confounding effects of reverse causality, thereby bolstering the robustness and credibility of our findings.

## Result

A total of 46, 7, 12, 20, 5, and 53 independent SNPs were found to be associated with hypothyroidism, hyperthyroidism, RA, T1D, MS, and overall autoimmune disease, respectively. These associations were established as statistically significant at a stringent threshold (p < 5.0*10^-8), ensuring a marked level of genetic distinction (r^2^ < 0.001). After eliminating SNPs related to potential confounders, palindromic SNPs, SNPs absent from the frailty-associated trait dataset and SNPs with conflicting alleles between exposure and outcome, 37 SNPs associated with hypothyroidism, 5 SNPs associated with hyperthyroidism, 7 SNPs associated with RA, 17 SNPs associated with T1D, 4 SNPs associated with MS, and 38 SNPs associated with overall autoimmune disease were included in this study.

**Figure 4:**
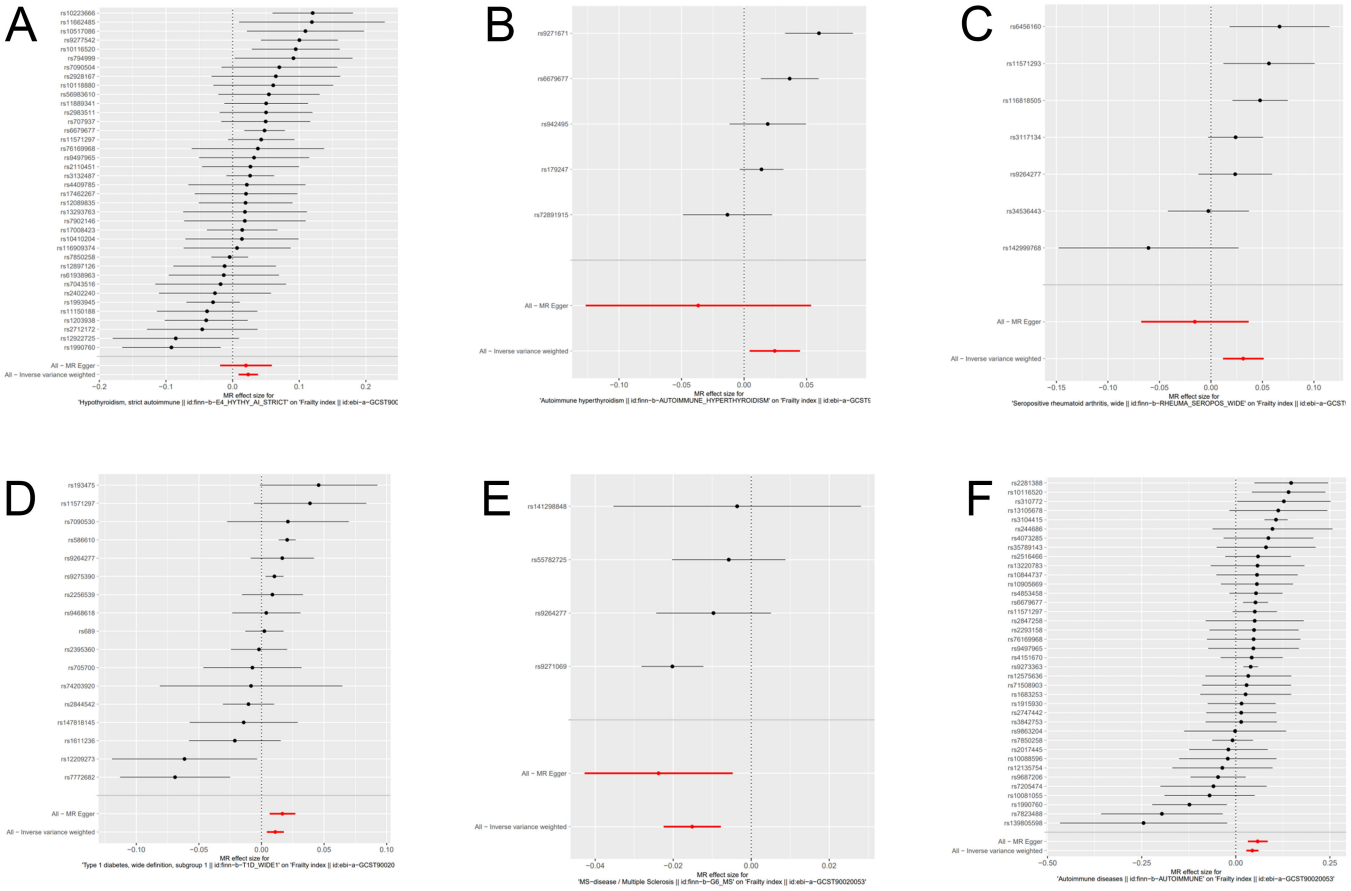
Forest plot of genetic causality between autoimmune diseases and frailty. **(A)** Hypothyroidism; **(B)** Hyperthyroidism; **(C)** Rheumatoid arthritis; **(D)** Type 1 diabetes; **(E)** Multiple sclerosis; **(F)** Overall autoimmune disease.

**Figure 5:**
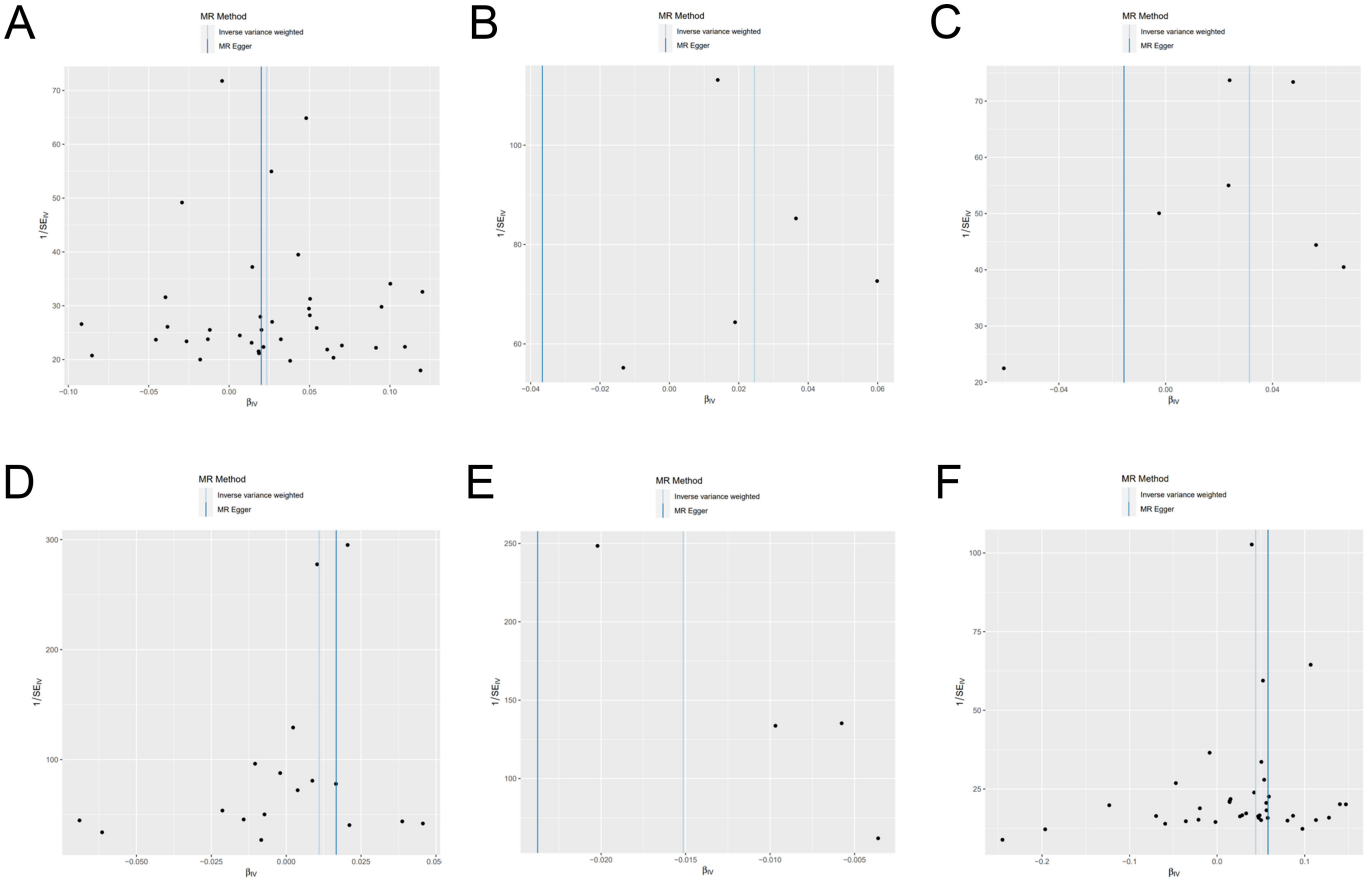
Funnel plot of genetic causality between autoimmune diseases and frailty. **(A)** Hypothyroidism; **(B)** Hyperthyroidism; **(C)** Rheumatoid arthritis; **(D)** Type 1 diabetes; **(E)** Multiple sclerosis; **(F)** Overall autoimmune disease.

**Figure 6:**
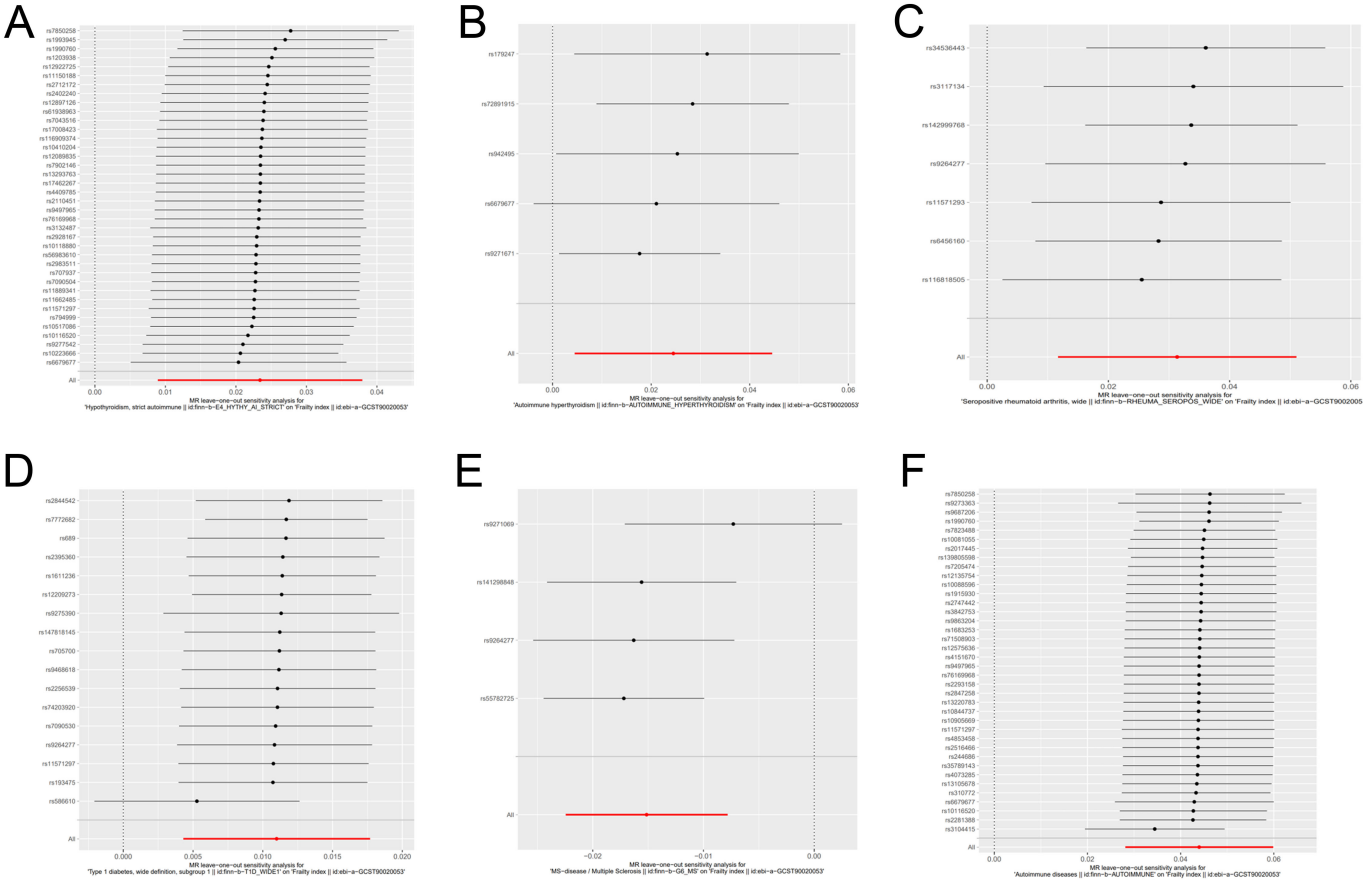
Forest plot for leave-one-out sensitivity analysis of genetic causality between autoimmune diseases and frailty. **(A)** Hypothyroidism; **(B)** Hyperthyroidism; **(C)** Rheumatoid arthritis; **(D)** Type 1 diabetes; **(E)** Multiple sclerosis; **(F)** Overall autoimmune disease.

We have generated six forest maps based on the final screened SNPs, which are elaborated upon in **Figure 4**. The specific information on excluded SNPs, along with the reasoning for their exclusion, is thoroughly documented in **Supplementary Table S3**. Furthermore, **Supplementary Tables S4–S9** provides comprehensive details of the SNPs included in the analysis. F-statistics for IVs linked to hypothyroidism, hyperthyroidism, RA, T1D, MS, and overall autoimmune disease were all > 10, suggesting significant associations between all IVs and exposure. As depicted in **Figure 5**, the funnel plots, which were derived from the IVW and MR-Egger analyses, demonstrate the absence of horizontal pleiotropy in the findings of all studies. Furthermore, the results of the “leave-one-out” assessments demonstrated that none of the SNPs had a notable impact on the IVW point estimates in any of the studies, as shown in **Figure 6**.

### Causal relationship between hypothyroidism and frailty

**Figure 7:**
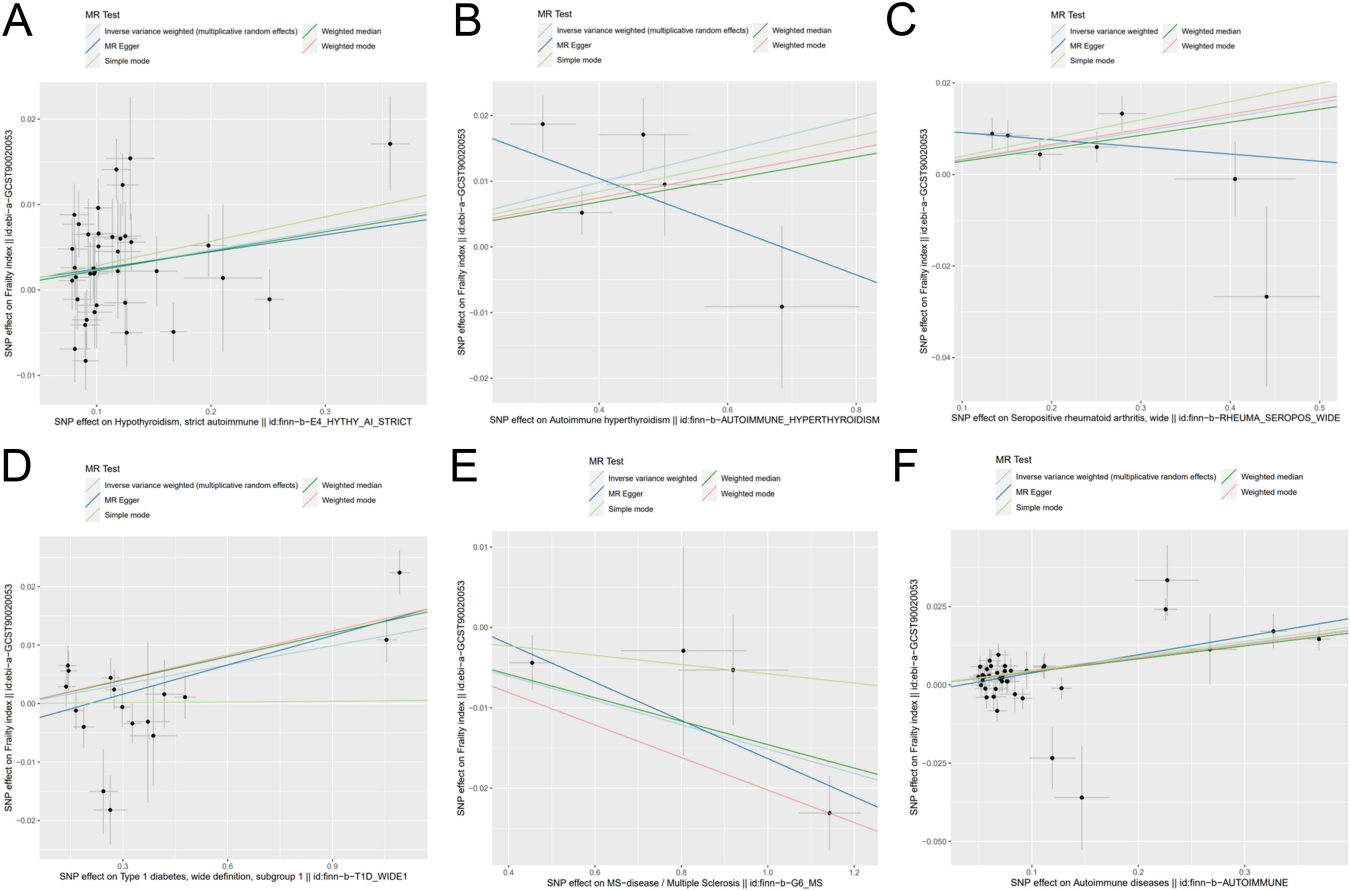
Scatter plot of genetic causality between autoimmune diseases and frailty using various MR methods. **(A)** Hypothyroidism; **(B)** Hyperthyroidism; **(C)** Rheumatoid arthritis; **(D)** Type 1 diabetes; **(E)** Multiple sclerosis; **(F)** Overall autoimmune disease.

As primary measure, IVW analysis (OR = 1.023, 95% CI: 1.008–1.038, p = 0.0015) clearly showed a statistically significant association between hypothyroidism and frailty (**Table 1**). Similarly, the Weighted Median method (OR = 1.022, 95% CI: 1.005–1.041, p = 0.0119) suggests that hypothyroidism is associated with a higher probability of frailty. However, neither the MR-Egger (OR = 1.020, 95% CI: 0.981–1.060, p = 0.3173), the Weighted Mode (OR = 1.028, 95% CI: 0.994–1.064, p = 0.1119), nor the Simple Mode (OR = 1.028, 95% CI: 0.990–1.069, p = 0.1519) methods proved that hypothyroidism and frailty are causally related. As illustrated in **Figure 7**, the five methods demonstrate correlation and direction. Hence, it can be inferred that hypothyroidism and frailty possess a significant correlation and that hypothyroidism increases the risk of frailty.

**Table 1:** Two-sample Mendelian randomized analyses for the associations of hypothyroidism/hyperthyroidism/rheumatoid arthritis/type 1 diabetes/multiple sclerosis/overall autoimmune disease with the risk of frailty.

Exposure	Outcome	method	SNP	Beta	OR (95%CI)	SE	P value
Hypothyroidism	Frailty index	Inverse variance weighted (multiplicative random effects)	37	0.0234	1.023(1.008-1.038)	0.0073	0.0015
		MR Egger	37	0.0199	1.020(0.981-1.060)	0.0196	0.3173
		Weighted median	37	0.0226	1.022(1.005-1.041)	0.0090	0.0119
		Weighted mode	37	0.0285	1.028(0.994-1.064)	0.0175	0.1119
		Simple mode	37	0.0285	1.028(0.990-1.069)	0.0194	0.1519
Hyperthyroidism	Frailty index	Inverse variance weighted (multiplicative random effects)	5	0.0245	1.024(1.004-1.045)	0.0102	0.0163
		MR Egger	5	-0.0366	0.964(0.881-1.054)	0.0459	0.4834
		Weighted median	5	0.0171	1.017(1.002-1.032)	0.0075	0.0233
		Weighted mode	5	0.0186	1.018(1.002-1.035)	0.0084	0.0923
		Simple mode	5	0.0210	1.021(0.995-1.047)	0.0128	0.1754
Rheumatoid arthritis	Frailty index	Inverse variance weighted	7	0.0313	1.031(1.011-1.052)	0.0100	0.0017
		MR Egger	7	-0.0156	0.984(0.934-1.037)	0.0265	0.5820
		Weighted median	7	0.0285	1.028(1.010-1.048)	0.0094	0.0024
		Weighted mode	7	0.0329	1.033(1.009-1.057)	0.0118	0.0316
		Simple mode	7	0.0397	1.040(1.012-1.069)	0.0139	0.0290
Type 1 Diabetes	Frailty index	Inverse variance weighted (multiplicative random effects)	17	0.0109	1.011(1.004-1.017)	0.0034	0.0012
		MR Egger	17	0.0167	1.016(1.006-1.027)	0.0051	0.0056
		Weighted median	17	0.0133	1.013(1.007-1.019)	0.0032	3.99E-05
		Weighted mode	17	0.0138	1.013(1.009-1.018)	0.0024	3.07E-05
		Simple mode	17	0.0004	1.000(0.988-1.012)	0.0063	0.9447
Multiple Sclerosis	Frailty index	Inverse variance weighted	4	-0.0151	0.984(0.977-0.992)	0.0037	4.87E-05
		MR Egger	4	-0.0237	0.976(0.958-0.995)	0.0096	0.1333
		Weighted median	4	-0.0145	0.985(0.978-0.992)	0.0037	8.97E-05
		Weighted mode	4	-0.0202	0.979(0.972-0.987)	0.0041	0.0161
		Simple mode	4	-0.0057	0.994(0.978-1.009)	0.0078	0.5175
Overall autoimmune disease	Frailty index	Inverse variance weighted (multiplicative random effects)	38	0.0439	1.044(1.028-1.061)	0.0080	5.32E-08
		MR Egger	38	0.0581	1.059(1.032-1.087)	0.0132	9.67E-05
		Weighted median	38	0.0414	1.042(1.024-1.060)	0.0087	2.23E-06
		Weighted mode	38	0.0430	1.043(1.025-1.062)	0.0091	3.43E-05
		Simple mode	38	0.0460	1.047(1.010-1.084)	0.0180	0.0148

SNP, single nucleotide polymorphism; OR, odds ratio; CI, confidence interval; SE, standard error (the standard error is an estimate).

As shown in **Tables 2**, **3**, quality control measures for this study encompassed rigorous evaluations for heterogeneity, horizontal polytropy, and sensitivity. Cochran’s Q test revealed notable heterogeneity (p = 4.04*10^-8), therefore we employed a random-effects IVW model. Based on the MR-Egger regression analysis, the results are unlikely to be biased by horizontal pleiotropy (intercept = 0.0004, p = 0.34). There were no significant outliers detected by the MR-PRESSO algorithm, and the results showed statistical significance (P_Raw_ = 0.0053).

**Table 2:** Heterogeneity and pleiotropy analysis by MR Egger, inverse variance weighted (IVW).

Exposure	Outcome	method	Cochran Q statistic	Heterogeneity p-value	Pleiotropy p-value	Pleiotropy SE	Egger Intercept
Hypothyroidism	Frailty index	MR Egger	115.53	4.04E-08	0.34	0.00	-0.00
		IVW	117.89	3.13E-08			
Hyperthyroidism	Frailty index	MR Egger	8.38	0.03	0.26	0.01	0.02
		IVW	13.55	0.00			
Rheumatoid Arthritis	Frailty index	MR Egger	110.72	0.20	0.12	0.00	0.01
		IVW	112.83	0.05			
Type 1 diabetes	Frailty index	MR Egger	38.01	8E-04	0.17	0.00	-0.00
		IVW	43.21	2E-04			
Multiple Sclerosis	Frailty index	MR Egger	2.88	0.23	0.43	0.00	0.00
		IVW	4.23	0.23			
Overall autoimmune diseases	Frailty index	MR Egger	72.65	0.00	0.18	0.00	-0.00
		IVW	76.27	0.00			

**Table 3:** Results of tests for MR-Pleiotropy RESidual sum and outlier.

Exposure	Outcome	*P*-value (Raw)	Outliers	*P*-value (Outlier-corrected)
Hypothyroidism	Frailty	0.0053	NA	NA
Hyperthyroidism	Frailty	0.0742	NA	NA
RA	Frailty	0.0203	NA	NA
T1D	Frailty	0.0010	rs586610; rs7772682	0.0095
MS	Frailty	0.0269	NA	NA
OAD	Frailty	3.59E-06	rs1990760; rs3104415	9.25E-06

RA, rheumatoid arthritis; MS, multiple sclerosis; T1D, type 1 diabetes; OAD, overall autoimmune disease.

### Causal relationship between hyperthyroidism and frailty

It was found that hyperthyroidism and frailty were statistically correlated both with IVW analysis (OR = 1.024, 95% CI: 1.004–1.045, p = 0.0163) and Weighted Median method (OR = 1.017, 95% CI: 1.002–1.032, p = 0.0233). **Figure 7** visually demonstrates that hyperthyroidism amplifies the propensity for developing frailty. Nonetheless, neither the MR-Egger (OR = 0.964, 95% CI: 0.881–1.054, p = 0.4834) nor the Weighted Mode (OR = 1.018, 95% CI: 1.002–1.035, p = 0.0923), nor the Simple Mode (OR = 1.021, 95% CI: 0.995–1.047, p = 0.1754) were able to establish a causal relationship between hyperthyroidism and frailty.

Similarly, Cochran’s Q test indicated heterogeneity (p = 0.0386), prompting the adoption of a random-effects IVW model. There was no horizontal pleiotropy found using MR-Egger regression analysis (intercept = 0.0250, p = 0.2672). Outliers were not identified in the MR-PRESSO analyses; however, their results did not display statistical significance (P_Raw_ = 0.0742).

### Causal relationship between rheumatoid arthritis and frailty

A significant association between frailty and RA was found from IVW analysis (OR = 1.031, 95% CI: 1.011–1.052, p = 0.0017), Weighted Median analysis (OR = 1.028, 95% CI: 1.010–1.048, p = 0.0024), weighted mode analysis (OR = 1.033, 95% CI: 1.009–1.057, p = 0.0316), and simple mode analysis (OR = 1.040, 95% CI: 1.012–1.069, p = 0.0290). **Figure 7** visually demonstrates that frailty can be exacerbated by RA. However, MR-Egger (OR =0.984, 95% CI: 0.934–1.037, p = 0.5820) could not prove causality.

As illustrated in **Tables 2**, **3**, there was no evidence of heterogeneity between the IV estimates derived from individual variants (p = 0.2016) when Cochran’s Q test was performed. Further analytic evidence supporting the absence of horizontal pleiotropy was provided by the MR-Egger regression (intercept = 0.0106, p = 0.1210). The MR-PRESSO analysis found no outliers, and the results were statistically significant (P_Raw_ = 0.0203).

### Causal relationship between type 1 diabetes and frailty

Based on IVW analysis (OR = 1.011, 95% CI: 1.004–1.017, p = 0.0012), MR Egger (OR = 1.016, 95% CI: 1.006–1.027, p = 0.0056), weighted median (OR = 1.013, 95% CI: 1.007–1.019, p = 3. 99*10^-5), and weighted mode (OR = 1.013, 95% CI: 1.009–1.018, p = 3. 07*10^-5), we can conclude that frailty and T1D are strongly associated, with the latter potentially exacerbating the former. **Figure 7** also illustrates the consistent results.

Cochran’s Q test unveiled considerable heterogeneity (p = 0.0008), necessitating the implementation of a random-effects IVW model. The results from the MR-Egger regression analyses indicated that the findings remained unaffected by horizontal pleiotropy (intercept = -0.0034, p = 0.1725). Despite the identification of outliers (rs586610; rs7772682) in the MR-PRESSO analyses, the results were still statistically significant before and after removing outliers (P_Raw_ = 0.0010, P_Outlier-corrected_ = 0.0095).

### Causal relationship between multiple sclerosis and frailty

The outcomes derived from the IVW analysis (OR = 0.984, 95% CI: 0.977–0.992, p = 4.87E-05), weighted median (OR = 0.985, 95% CI: 0.978–0.992, p = 8.97*10^-5), and weighted mode (OR = 0.979, 95% CI: 0.972–0.987, p = 0.0161) collectively suggest a robust association between frailty and MS. It should be noted that, as depicted in **Figure 7**, MS mitigates the risk of developing frailty. The MR-Egger method (OR = 0.976, 95% CI: 0.958–0.995, p = 0.1333) and the Simple mode method (OR = 0.994, 95% CI: 0.978–1.009, p = 0.5175) did not yield definitive evidence of causality.

There was no significant heterogeneity in the data (p = 0.2360) according to Cochran’s Q test. According to the MR-Egger regression analysis, horizontal pleiotropy was not present (intercept = 0.0074, p = 0.4353). No outliers were found in the MR-PRESSO analysis, and the results obtained by the algorithm were statistically significant (P_Raw_ = 0.0269).

### Causal relationship between overall autoimmune disease and frailty

Five algorithms, encompassing IVW analysis (OR = 1.044, 95% CI: 1.028–1.061, p = 5.32*10^-8), MR Egger (OR = 1.059, 95% CI: 1.032–1.087, p = 9.67*10^-5), weighted median (OR = 1.042, 95% CI: 1.024–1.060, p = 2.23*10^-6), weighted mode (OR = 1.043, 95% CI: 1.025–1.062, p = 3.43*10^-5), and simple mode (OR = 1.047, 95% CI: 1.010–1.084, p = 0.0148) consistently yielded results demonstrating a substantial correlation between overall autoimmune disease and frailty. Besides, **Figure 7** visually depicts that overall autoimmune disease escalates the likelihood of developing frailty.

Due to the notable diversity observed, as indicated by Cochran’s Q test (p = 0.0002), it was imperative to employ a random-effects IVW model. The MR-Egger regression analysis provides compelling evidence that the result is improbable to be influenced by horizontal pleiotropy (intercept = -0.0020, p = 0.1891). Identified outliers, namely rs1990760 and rs3104415, in the MR-PRESSO analyses, did not significantly alter the statistical significance of the results, which remained consistent even after their removal (P_Raw_ = 3.59*10^-6, P_Outlier-corrected_ = 9.25*10^-6).

### Reverse MR analysis

During the reverse MR study, while utilizing hypothyroidism, RA, MS, and overall autoimmune disease as outcome variables, we noted the presence of horizontal pleiotropy (Hypothyroidism: Ppleiotropy = 5.14e-05; RA: Ppleiotropy = 0.000; MS: Ppleiotropy = 0.001; Overall autoimmune disease: Ppleiotropy = 0.000). Furthermore, the IVW values did not attain statistical significance when incorporating hyperthyroidism and T1D as outcome variables. Therefore, it can be inferred that frailty does not play a contributory role in the initiation and progression of these autoimmune diseases.

## Discussion

Utilizing two-sample MR analysis, we substantiated the causal impact of both five common autoimmune diseases and overall autoimmune disease on frailty. Horizontal pleiotropy manifests when a genetic variation exhibits associations with diverse phenotypes through distinct pathways, potentially compromising the reliability of MR analysis. Through a meticulous screening of SNPs and computational analyses of IVs, it was established that hypothyroidism, hyperthyroidism, RA, T1D, and overall autoimmune disease had a positive causal correlation with frailty, while the causal relationship between MS and frailty was negative. Furthermore, our investigation has revealed that this study is the pioneering application of MR Horizontal pleiotropy manifests when a genetic variation exhibits associations with diverse phenotypes through distinct pathways, potentially compromising the reliability of MR analysis methods to demonstrate the underlying causal connection between autoimmune disease and frailty. Ultimately, inverse MR analyses have corroborated the absence of reverse causality between these autoimmune diseases and frailty.

Multiple clinical studies have validated a notable correlation between frailty and an escalation in morbidity, mortality, hospitalization, instances of falls, and the requirement for extended care (3, 9). Peter et al. meticulously tracked a cohort of 493,737 participants over seven years, unveiling that 72% of frail patients have multimorbidity, with 27% experiencing at least four long-term illnesses (9). Furthermore, it was ascertained that frail individuals aged over 45 faced a risk of mortality more than twofold higher than their non-frail counterparts. The prevalence of frailty has been demonstrated to be 53% among long-term care inpatients, 42% among patients with actual or malignant hematological disease, and 37% among patients with end-stage renal disease, according to various systematic reviews (23–25). The prevalence and deleterious implications of frailty markedly augment the economic burden on healthcare for society. In a prospective observational study, Robinson et al. identified an additional cost of $48,632 incurred by frail patients six months after surgical intervention (26). Kristine et al. conducted a prospective cohort study, revealing that frail elderly women incurred an additional annual sum of $6,974 in total healthcare expenditures in comparison to their robust counterparts (27). Hence, physicians must accord heightened consideration to the prevention and adept management of frailty in the therapeutic interventions for specific associated maladies. This strategic approach not only expedites the early convalescence of patients following surgical procedures, but also alleviates the financial burden on healthcare systems.

Currently, there exists a paucity of investigations delving into the intricate correlation between hypothyroidism and frailty. Bo et al. discovered a significant association between subclinical hypothyroidism and frailty (OR =2.18) through meticulous logistic regression analysis (5). This study aligns harmoniously with our discovery, affirming that hypothyroidism catalyzes the promotion of frailty. Beatrice et al. observed a consistent trend among centenarians, wherein free triiodothyronine (FT3) and thyroid stimulating hormone exhibited a negative correlation with frailty, while free thyroxine (FT4) demonstrated a positive association with frailty (28). Diminished levels of FT3 influence myosin expression, culminating in the depletion of muscle mass and strength, thereby potentially precipitating the onset of frailty (29, 30).

In a prospective cohort study, it emerged that male participants manifesting subclinical hyperthyroidism were predisposed to a frailty state at a rate 2.5 times higher than their normal counterparts, with particular emphasis on those under the age of 74 (OR for frailty = 3.63) (6). Yeap’s cross-sectional Australian study establishes a noteworthy correlation, revealing that elevated levels of FT4 are linked to an increased risk of frailty in men aged 70–89 years (31). Our findings align with the outcomes of these studies, further supporting the claim that hyperthyroidism indeed contributes to the progression of frailty. However, a limitation of our analysis lies in the relatively scant inclusion of IVs. Thus, the imperative for well-conceived prospective studies and additional MR horizontal pleiotropy manifests when a genetic variation exhibits associations with diverse phenotypes through distinct pathways, potentially compromising the reliability of MR analysis in the future remains, to substantiate this observed correlation more comprehensively.

With the upward trajectory of life expectancy, a projection indicates the emergence of 800,000 RA patients in Japan, with a substantial two-thirds of this demographic cohort surpassing the age of 65 (32). A prospective observational study conducted by Tada revealed that frailty was present in only 6.7% of patients in remission from RA, while it affected 46.7% of patients with moderately and highly active RA (33). Hence, a discernible correlation exists between frailty and disease activity in RA, underscoring the pivotal significance of effectively managing RA disease activity as a preventive measure against frailty. Employing the Fried phenotype as a metric, Michael et al. scrutinized a cohort of 457,561 patients for frailty, revealing an overall incidence of 3.4% in the entire cohort, with a notably higher prevalence of 18.6% among individuals with RA (34). Similarly, the evidence presented in this study buttresses the substantial correlation between RA and an elevated prevalence of frailty, elucidating a positive causal relationship between the two. Furthermore, RA engenders a substantial release of pro-inflammatory cytokines, prominently including IL-6, TNF-α, and C-reactive protein (4). Heightened concentrations of these pro-inflammatory cytokines exhibit a robust association with frailty, functional decline, and loss of muscle mass (35). The discernment of a correlation between RA and frailty holds paramount significance, as it provides a pivotal avenue for directing potential preventive measures aimed at mitigating the onset of frailty.

Although epidemiological investigations validating the correlation between T1D and frailty are lacking, certain studies suggest that T1D might exert an indirect influence on frailty through various mechanisms. Several studies have elucidated that children with T1D exhibit decreased muscle strength and power, coupled with an increased susceptibility to fatigue when contrasted with their non-diabetic counterparts (36, 37). In comparison to the control group, Jakobsen et al. observed a significant reduction in fiber diameter in the tibialis anterior muscle of patients with T1D (61.8 microns versus 77.8 microns) (38). We found a significant association between T1D and frailty in our study, with MR analysis revealing a promotional effect of T1D on frailty development. Moreover, Andreassen et al. observed that neuropathic RA patients exhibited a remarkable reduction in calf muscle volume exceeding 50% in 13 years, while non-neuropathic T1D patients experienced a comparatively milder reduction of only 20% in calf muscle volume (39). This implies that T1D might indirectly contribute to frailty by instigating neurological lesions that culminate in neuromuscular atrophy. Lastly, in a mouse model of T1D, Donna et al. discovered that sustained activation of the Notch signaling pathway in satellite cells impeded their capacity to transition from quiescence to proliferation (40). Therefore, T1D not only reduces the quantity of these muscle stem cells but also undermines their effectiveness in initiating the reparative process, ultimately contributing to the development of frailty.

A recent investigation conducted by the UK Biobank has conclusively shown that MS is the chronic ailment displaying the most robust correlation with frailty, and MS patients were found to exhibit a 15-fold heightened risk of developing frailty (9). Utilizing the Tilburg Frailty Index (TFI) scale, Frau et al. discovered that a substantial 62.5% of individuals with MS exhibited frailty, with an average TFI score of merely 5.7 (41). In an expansive and scrupulously controlled longitudinal investigation, Cortese et al. discerned that the age-related decline in physical functioning is accelerated by 15–30 years in women navigating the aging continuum with MS (42). While our findings indicate a robust association between MS and frailty, it should be noted that all MR algorithms employed in our study consistently suggest that MS, counterintuitively, diminishes the risk of frailty. This discrepancy in our findings, suggesting a potential protective effect of MS on frailty in the context of MR, could indeed be linked to the limited number of IVs incorporated in our study. In the future, we may be able to gain a deeper understanding of the relationship between MS and frailty by a more extensive set of IVs. However, MS is prevalent among younger individuals. Furthermore, a recent two-sample MR analysis revealed a positive causal relationship between leukocyte telomere length and MS, which lends support to the hypothesis that MS impedes the development of frailty (43).

This study’s main strength is its innovative use of MR analysis to confirm the causal relationship between five common autoimmune diseases and overall autoimmune disease in frail individuals. MR serves to mitigate potential biases inherent in observational studies by employing IVs related to the exposure as proxies for the exposure itself. This approach effectively severs the inherent connection between autoimmune disease-associated alleles and lifestyle or demographic variables, thereby mitigating the potential distortion in the relationship between autoimmune disease and frailty.

This study necessitates the acknowledgment of several inherent limitations. Firstly, it is imperative to underscore that our study was conducted within the confines of a European population. It is crucial to recognize that ethnicity and the potential for selection bias may indirectly impact the causal inferences derived from the study. Secondly, frailty evaluation was mainly based on the frailty index, a subjective metric. The absence of objective indicators, such as telomere length or genomic DNA damage, in this analysis, underscores the need for further exploration to gain a more comprehensive understanding of frailty within the context of autoimmune disease. Thirdly, this study is deficient in pharmacogenomic analyses for predictive therapeutic strategies in the context of these autoimmune diseases. Murdaca et al. employed pharmacogenomics to anticipate that etanercept might demonstrate advantageous efficacy in addressing psoriasis and psoriatic arthritis (44). Given its role as a TNF-α inhibitor, etanercept potentially holds significant advantages for treating autoimmune disorders. Consequently, future predictive analyses will likely necessitate the integration of pharmacogenomic methodologies. Fourthly, our study utilized summary-level data, making it impossible to conduct subgroup analyses by age or gender. Fifthly, MR analysis using genetic variants as IVs exclusively reveals the genetic component of a trait without accounting for environmental influences. The relatively modest causal effect of autoimmune disease on frailty observed in our study may be explained by this limitation.

## Conclusion

In summary, the current MR study provides evidence supporting a causal relationship between hypothyroidism, hyperthyroidism, rheumatoid arthritis, type 1 diabetes, multiple sclerosis, and overall autoimmune disease with frailty. Hypothyroidism, hyperthyroidism, rheumatoid arthritis, type 1 diabetes, and overall autoimmune disease were associated with an increased risk of frailty in our study. However, multiple sclerosis appeared to be linked to a potential reduction in the risk of frailty. Therefore, orthopedic surgeons should prioritize the identification of signs of frailty during the diagnosis and treatment of hypothyroidism, hyperthyroidism, rheumatoid arthritis, type 1 diabetes, and multiple sclerosis. This proactive approach has the potential to alleviate the economic burden on healthcare for both society and families.

## Data Availability

The original contributions presented in the study are included in the article/**Supplementary Material**. Further inquiries can be directed to the corresponding authors.
