# Supplementary material for: Does autoimmune diseases increase the risk of frailty? A Mendelian randomization study
Source: Front Endocrinol (Lausanne). 2024 Aug 27;15:1364368. doi: 10.3389/fendo.2024.1364368 (PMC11384993; doi:10.3389/fendo.2024.1364368)
Supplement: Supplementary file 1 [file Table1.doc]

| **Supplementary Table 1.** The 45 autoimmune diseases included in overall autoimmune disease. |
| --- |
| **Diseases** |
| Drug-induced autoimmune haemolytic anaemia |
| Other autoimmune haemolytic anaemias |
| Myasthenia gravis |
| Other demyelinating diseases of the central nervous system |
| Disorders of myoneural junction and muscle in diseases classified elsewhere |
| Coeliac disease |
| Primary biliary cholangitis |
| Guillain-Barre syndrome |
| Psoriasis |
| Vitiligo |
| Alopecia areata |
| Idiopathic thrombocytopenic purpura |
| Henoch-Schönlein purpura nephritis (HSPN) |
| Adrenocortical insufficiency |
| Hypersensitivity angiitis |
| IgA nephropathy (IgA glomerulonephritis) |
| Vitamin B12 deficiency anaemia |
| Anterior Iridocyclitis |
| Graves ophthalmopathy, strict |
| Bechet disease |
| Pemphigoid |
| Dermatitis herpetiformis |
| Mixed connective tissue disease |
| Rheumatoid arthritis |
| Systemic lupus erythematosus |
| Multiple Sclerosis |
| Type 1 diabetes |
| Inflammatory bowel disease |
| Relapsing polychondritis |
| Sicca syndrome |
| Systemic sclerosis |
| Dermatopolymyositis |
| Wegener granulomatosis |
| Microscopic polyangiitis |
| Polyarteritis with lung involvement |
| Allergic purpura |
| Rheumatic fever incl heart disease |
| Hypothyroidism, strict autoimmune |
| Acute disseminated encephalitis/encephalomyelitis |
| Other acute disseminated demyelination |
| Narcolepsy and cataplexy |
| Autoimmune hyperthyroidism |
| Autoimmune thyroiditis |
| Autoimmune polyglandular failure |
| Autoimmune hepatitis |

| **Supplementary Table 2.** The sources and corresponding information on exposures and outcomes. | | | |
| --- | --- | --- | --- |
| Traits | Population | Sample size | Consortium |
| Hypothyroidism (exposures) | European | 22,997 cases/175,475 controls | FinnGen/GWAS ID: finn-b-E4_HYTHY_AI_STRICT |
| Hyperthyroidism (exposures) | European | 962 cases/172,976 controls | FinnGen/GWAS ID: finn-b-AUTOIMMUNE_HYPERTHYROIDISM |
| RA (exposures) | European | 4,594 cases/214,196 controls | FinnGen/GWAS ID: finn-b-RHEUMA_SEROPOS_WIDE |
| T1D (exposures) | European | 4,849 cases/180,722 controls | FinnGen/GWAS ID: finn-b-T1D_WIDE1 |
| MS (exposures) | European | 1,048 cases/217,141 controls | FinnGen/GWAS ID: finn-b-G6_MS |
| Overall autoimmune disease (exposures) | European | 42,202 cases/176,590 controls | FinnGen/GWAS ID: finn-b-AUTOIMMUNE |
| Frailty (outcomes) | European | Total 175,226 individuals | UK Biobank/ GWAS ID: ebi-a-GCST90020053 |
| Abbreviations: RA, rheumatoid arthritis; T1D, type 1 diabetes; MS, multiple sclerosis. | | | |

| **Supplementary Table 3.** Excluded SNPs and the reasons for removing these SNPs. | | |
| --- | --- | --- |
| Traits | Excluded SNPs | Reasons for exclusion |
| Hypothyroidism | rs9273400 | Associated with MS, RA, T1D, etc. |
| Hypothyroidism | rs7310615 | Associated with smoking. |
| Hypothyroidism | rs3132487 | SNP with inconsistent alleles (T/G vs A/G) |
| Hypothyroidism | rs17786733 | Palindromic SNP |
| Hypothyroidism | rs7754251 | Palindromic SNP |
| Hypothyroidism | rs9842232 | Palindromic SNP |
| Hypothyroidism | rs201121732 | Not present in any of the frailty-related trait datasets |
| Hypothyroidism | rs5845323 | Not present in any of the frailty-related trait datasets |
| Hypothyroidism | rs78765971 | Not present in any of the frailty-related trait datasets |
| Hyperthyroidism | rs9275576 | Associated with depression, T1D, etc. |
| Hyperthyroidism | rs9265890 | Palindromic SNP |
| RA | rs6679677 | Associated with Hypothyroidism. |
| RA | rs2523572 | Associated with MS. |
| RA | rs3778754 | Palindromic SNP |
| RA | rs147415887 | Not present in any of the frailty-related trait datasets |
| RA | rs34434863 | Not present in any of the frailty-related trait datasets |
| T1D | rs6679677 | Associated with Hypothyroidism, RA. |
| T1D | rs28744290 | Associated with RA. |
| T1D | rs3184504 | Associated with Hypothyroidism, smoking. |
| OAD | rs3184504 | Associated with smoking. |
| OAD | rs7002588 | SNP with inconsistent alleles (A/C vs A/G) |
| OAD | rs11755527 | Palindromic SNP |
| OAD | rs17786733 | Palindromic SNP |
| OAD | rs2757041 | Palindromic SNP |
| OAD | rs34536443 | Palindromic SNP |
| OAD | rs3809822 | Palindromic SNP |
| OAD | rs6828814 | Palindromic SNP |
| OAD | rs932036 | Palindromic SNP |
| OAD | rs111352543 | Not present in any of the frailty-related trait datasets |
| OAD | rs201121732 | Not present in any of the frailty-related trait datasets |
| OAD | rs35508742 | Not present in any of the frailty-related trait datasets |
| OAD | rs5845323 | Not present in any of the frailty-related trait datasets |
| OAD | rs73188832 | Not present in any of the frailty-related trait datasets |
| OAD | rs78765971 | Not present in any of the frailty-related trait datasets |
| Abbreviations: RA, rheumatoid arthritis; MS, multiple sclerosis; T1D, type 1 diabetes; OAD, overall autoimmune disease; SNP, single-nucleotide polymorphism. | | |

**Supplementary Table 4.** Detailed Description of 37 SNPs Associated with Frailty in Hypothyroidism.

| SNP ID | A1 | A2 | Exposure (Hypothyroidism) | | | | |  | Outcome (Frailty) | | | |  |
| --- | --- | --- | --- | --- | --- | --- | --- | --- | --- | --- | --- | --- | --- |
|  |  |  | P value | Beta | SE | EAF | F-statistic |  | P value | Beta | SE | EAF |  |
| rs10116520 | G | A | 7.79E-18 | 0.1013 | 0.0118 | 0.4006 | 73.69718613 |  | 0.00463298 | 0.0096 | 0.0034 | 0.3855 |  |
| rs10118880 | A | G | 1.01E-09 | -0.0787 | 0.0129 | 0.7236 | 37.21911774 |  | 0.1787 | -0.0048 | 0.0036 | 0.6834 |  |
| rs10223666 | C | G | 7.53E-21 | 0.1173 | 0.0125 | 0.681 | 88.05865104 |  | 8.97E-05 | 0.0141 | 0.0036 | 0.694 |  |
| rs10410204 | T | C | 1.17E-11 | 0.0786 | 0.0116 | 0.5398 | 45.91188709 |  | 0.745501 | 0.0011 | 0.0034 | 0.5831 |  |
| rs10517086 | A | G | 3.29E-10 | 0.0805 | 0.0128 | 0.2815 | 39.55194558 |  | 0.0138299 | 0.0088 | 0.0036 | 0.3021 |  |
| rs11150188 | G | A | 3.95E-13 | -0.0913 | 0.0126 | 0.308 | 52.50449611 |  | 0.3266 | 0.0035 | 0.0035 | 0.3275 |  |
| rs11571297 | C | T | 1.20E-27 | -0.1303 | 0.012 | 0.3725 | 117.902325 |  | 0.0883995 | -0.0056 | 0.0033 | 0.486 |  |
| rs11662485 | A | G | 8.77E-10 | 0.1294 | 0.0211 | 0.08102 | 37.60968293 |  | 0.0322998 | 0.0154 | 0.0072 | 0.0558 |  |
| rs116909374 | T | C | 6.56E-10 | -0.2105 | 0.0341 | 0.03107 | 38.1058341 |  | 0.874 | -0.0014 | 0.0086 | 0.0392 |  |
| rs11889341 | T | C | 7.48E-20 | 0.1251 | 0.0137 | 0.2303 | 83.38146381 |  | 0.1105 | 0.0063 | 0.004 | 0.2233 |  |
| rs1203938 | A | G | 1.21E-18 | 0.1263 | 0.0143 | 0.7924 | 78.00647554 |  | 0.2169 | -0.005 | 0.004 | 0.7842 |  |
| rs12089835 | T | C | 3.55E-15 | 0.0978 | 0.0124 | 0.3173 | 62.20572689 |  | 0.589299 | 0.0019 | 0.0035 | 0.3576 |  |
| rs12897126 | A | T | 6.30E-12 | -0.125 | 0.0182 | 0.886 | 47.17080416 |  | 0.756 | 0.0015 | 0.0049 | 0.8666 |  |
| rs12922725 | G | A | 8.21E-10 | -0.0809 | 0.0132 | 0.7271 | 37.5616975 |  | 0.08 | 0.0069 | 0.0039 | 0.752 |  |
| rs13293763 | C | G | 2.44E-11 | 0.1185 | 0.0177 | 0.1198 | 44.82148054 |  | 0.6931 | 0.0022 | 0.0056 | 0.0977 |  |
| rs17008423 | T | C | 1.88E-17 | -0.1525 | 0.0179 | 0.1227 | 72.58212107 |  | 0.598 | -0.0022 | 0.0041 | 0.2014 |  |
| rs17462267 | C | A | 7.95E-14 | 0.0944 | 0.0126 | 0.2956 | 56.13050227 |  | 0.610401 | 0.0019 | 0.0037 | 0.277 |  |
| rs1990760 | T | C | 1.22E-14 | 0.0904 | 0.0117 | 0.5843 | 59.6981905 |  | 0.01438 | -0.0083 | 0.0034 | 0.6087 |  |
| rs1993945 | T | A | 4.16E-46 | 0.1672 | 0.0117 | 0.4089 | 204.2193327 |  | 0.154 | -0.0049 | 0.0034 | 0.3801 |  |
| rs2110451 | A | G | 4.84E-14 | 0.0972 | 0.0129 | 0.2775 | 56.77395371 |  | 0.4709 | 0.0026 | 0.0036 | 0.2978 |  |
| rs2402240 | A | G | 9.21E-11 | 0.0982 | 0.0152 | 0.1759 | 41.73801874 |  | 0.5438 | -0.0026 | 0.0042 | 0.1911 |  |
| rs2712172 | A | G | 5.55E-14 | -0.09 | 0.012 | 0.3708 | 56.24948581 |  | 0.2754 | 0.0041 | 0.0038 | 0.2612 |  |
| rs2928167 | G | A | 2.70E-12 | -0.1017 | 0.0145 | 0.1993 | 49.19284402 |  | 0.1858 | -0.0066 | 0.005 | 0.1273 |  |
| rs2983511 | C | G | 9.59E-15 | -0.1016 | 0.0131 | 0.2626 | 60.15072339 |  | 0.1551 | -0.0051 | 0.0036 | 0.3048 |  |
| rs4409785 | C | T | 1.94E-10 | 0.0983 | 0.0154 | 0.1665 | 40.7438087 |  | 0.624001 | 0.0021 | 0.0044 | 0.1717 |  |
| rs56983610 | A | C | 4.68E-11 | -0.1138 | 0.0173 | 0.1299 | 43.27014474 |  | 0.1634 | -0.0062 | 0.0044 | 0.1672 |  |
| rs61938963 | T | C | 1.68E-11 | 0.0832 | 0.0124 | 0.3188 | 45.01935954 |  | 0.7604 | -0.0011 | 0.0035 | 0.3432 |  |
| rs6679677 | A | C | 1.64E-102 | 0.3567 | 0.0166 | 0.1446 | 461.7278521 |  | 0.001736 | 0.0171 | 0.0055 | 0.1018 |  |
| rs7043516 | C | A | 1.60E-10 | -0.1 | 0.0156 | 0.1676 | 41.09101162 |  | 0.7208 | 0.0018 | 0.005 | 0.1219 |  |
| rs707937 | G | C | 1.27E-16 | -0.1208 | 0.0146 | 0.1994 | 68.45799684 |  | 0.1427 | -0.006 | 0.0041 | 0.1995 |  |
| rs7090504 | A | T | 8.14E-12 | 0.0927 | 0.0136 | 0.2379 | 46.45983698 |  | 0.1146 | 0.0065 | 0.0041 | 0.202 |  |
| rs76169968 | A | G | 1.20E-09 | -0.1186 | 0.0195 | 0.09875 | 36.99100966 |  | 0.4507 | -0.0045 | 0.006 | 0.0835 |  |
| rs7850258 | G | A | 1.44E-93 | 0.2512 | 0.0122 | 0.6544 | 423.9509754 |  | 0.7563 | -0.0011 | 0.0035 | 0.6684 |  |
| rs7902146 | T | C | 8.58E-10 | -0.0818 | 0.0133 | 0.7506 | 37.82677842 |  | 0.685499 | -0.0015 | 0.0038 | 0.746 |  |
| rs794999 | G | A | 8.23E-10 | -0.0843 | 0.0137 | 0.7727 | 37.86256614 |  | 0.0431996 | -0.0077 | 0.0038 | 0.7487 |  |
| rs9277542 | C | T | 3.76E-19 | -0.1227 | 0.0137 | 0.2539 | 80.21286365 |  | 0.0005868 | -0.0123 | 0.0036 | 0.3041 |  |
| rs9497965 | T | C | 4.07E-11 | 0.0808 | 0.0122 | 0.3367 | 43.8630766 |  | 0.4345 | 0.0026 | 0.0034 | 0.4086 |  |

SNP: single nucleotide polymorphism; A1: effect allele; A2: other allele; SE: standard error; EAF: effect allele frequency.

**Supplementary Table 5.** Detailed Description of 5 SNPs Associated with Frailty in Hyperthyroidism.

| SNP ID | A1 | A2 | Exposure (Hyperthyroidism) | | | | |  | Outcome (Frailty) | | | |
| --- | --- | --- | --- | --- | --- | --- | --- | --- | --- | --- | --- | --- |
|  |  |  | P value | Beta | SE | EAF | F-statistic |  | P value | Beta | SE | EAF |
| rs179247 | G | A | 2.47E-15 | -0.3733 | 0.0472 | 0.5785 | 62.54995317 |  | 0.1144 | -0.0052 | 0.0033 | 0.4817 |
| rs6679677 | A | C | 1.20E-11 | 0.4689 | 0.0692 | 0.1402 | 45.91380473 |  | 0.001736 | 0.0171 | 0.0055 | 0.1018 |
| rs72891915 | A | G | 1.31E-08 | 0.6845 | 0.1204 | 0.03931 | 32.32130872 |  | 0.4621 | -0.0091 | 0.0124 | 0.0191 |
| rs9271671 | T | G | 8.19E-10 | -0.3124 | 0.0509 | 0.5815 | 37.66877457 |  | 1.13E-05 | -0.0187 | 0.0043 | 0.1826 |
| rs942495 | T | C | 2.75E-08 | 0.502 | 0.0903 | 0.07115 | 30.90487134 |  | 0.2234 | 0.0095 | 0.0078 | 0.0495 |

SNP: single nucleotide polymorphism; A1: effect allele; A2: other allele; SE: standard error; EAF: effect allele frequency

**Supplementary Table 6.** Detailed Description of 7 SNPs Associated with Frailty in Rheumatoid Arthritis.

| SNP ID | A1 | A2 | Exposure (Hypothyroidism) | | | | |  | Outcome (Frailty) | | | |
| --- | --- | --- | --- | --- | --- | --- | --- | --- | --- | --- | --- | --- |
|  |  |  | P value | Beta | SE | EAF | F-statistic |  | P value | Beta | SE | EAF |
| rs11571293 | T | G | 3.47E-10 | -0.151 | 0.0241 | 0.3158 | 39.2568 |  | 0.0110 | -0.008 | 0.0034 | 0.4074 |
| rs116818505 | G | T | 1.29E-24 | -0.2789 | 0.0272 | 0.2559 | 105.1369 |  | 0.0004 | -0.013 | 0.0038 | 0.2789 |
| rs142999768 | T | C | 1.28E-13 | 0.4406 | 0.0595 | 0.03863 | 54.8341 |  | 0.1736 | -0.026 | 0.0196 | 0.0086 |
| rs3117134 | C | T | 1.71E-27 | -0.2506 | 0.0231 | 0.5154 | 117.6885 |  | 0.0763 | -0.006 | 0.0034 | 0.376 |
| rs34536443 | C | G | 1.79E-09 | -0.4054 | 0.0674 | 0.0304 | 36.1779 |  | 0.9008 | 0.001 | 0.0081 | 0.046 |
| rs6456160 | C | T | 3.03E-09 | -0.1336 | 0.0225 | 0.5583 | 35.2568 |  | 0.0073 | -0.008 | 0.0033 | 0.5631 |
| rs9264277 | C | T | 6.46E-13 | 0.187 | 0.026 | 0.73 | 51.7288 |  | 0.1965 | 0.004 | 0.0034 | 0.633 |

SNP: single nucleotide polymorphism; A1: effect allele; A2: other allele; SE: standard error; EAF: effect allele frequency.

**Supplementary Table 7.** Detailed Description of 17 SNPs Associated with Frailty in Type 1 Diabetes.

| SNP ID | A1 | A2 | Exposure (Hypothyroidism) | | | | |  | Outcome (Frailty) | | | |
| --- | --- | --- | --- | --- | --- | --- | --- | --- | --- | --- | --- | --- |
|  |  |  | P value | Beta | SE | EAF | F-statistic |  | P value | Beta | SE | EAF |
| rs11571297 | C | T | 1.43E-09 | -0.1445 | 0.0239 | 0.3708 | 36.5540256 |  | 0.0883995 | -0.0056 | 0.0033 | 0.486 |
| rs12209273 | C | G | 1.78E-09 | 0.244 | 0.0406 | 0.08422 | 36.1179344 |  | 0.0370297 | -0.015 | 0.0072 | 0.0584 |
| rs147818145 | A | C | 8.50E-09 | 0.3872 | 0.0672 | 0.03071 | 33.19918868 |  | 0.5128 | -0.0055 | 0.0085 | 0.0408 |
| rs1611236 | A | G | 5.13E-11 | -0.1878 | 0.0286 | 0.2235 | 43.11758508 |  | 0.2612 | 0.004 | 0.0035 | 0.3202 |
| rs193475 | C | T | 7.63E-09 | -0.1424 | 0.0246 | 0.6835 | 33.5077359 |  | 0.0571597 | -0.0065 | 0.0034 | 0.6135 |
| rs2256539 | T | C | 1.74E-28 | 0.2747 | 0.0248 | 0.6678 | 122.6900311 |  | 0.4881 | 0.0024 | 0.0034 | 0.611 |
| rs2395360 | A | C | 4.93E-35 | -0.2983 | 0.0242 | 0.6187 | 151.9396404 |  | 0.8609 | 6.00E-04 | 0.0034 | 0.5949 |
| rs2844542 | G | C | 1.11E-39 | 0.3272 | 0.0248 | 0.3397 | 174.067843 |  | 0.311 | -0.0034 | 0.0034 | 0.4093 |
| rs586610 | C | T | 1.00E-200 | 1.0921 | 0.0293 | 0.2086 | 1389.264355 |  | 1.39E-09 | 0.0224 | 0.0037 | 0.3029 |
| rs689 | T | A | 6.87E-55 | 0.4779 | 0.0306 | 0.792 | 243.9082709 |  | 0.772999 | 0.0011 | 0.0037 | 0.7104 |
| rs705700 | C | T | 2.80E-12 | 0.1655 | 0.0237 | 0.3866 | 48.76347238 |  | 0.728501 | -0.0012 | 0.0033 | 0.4231 |
| rs7090530 | A | C | 3.14E-08 | 0.1374 | 0.0248 | 0.6752 | 30.69484348 |  | 0.3955 | 0.0029 | 0.0034 | 0.6065 |
| rs74203920 | T | C | 8.60E-10 | 0.3717 | 0.0606 | 0.03724 | 37.62142082 |  | 0.8229 | -0.0031 | 0.0138 | 0.0153 |
| rs7772682 | A | T | 3.53E-08 | -0.2637 | 0.0478 | 0.0669 | 30.43405252 |  | 0.00183202 | 0.0182 | 0.0059 | 0.0866 |
| rs9264277 | C | T | 2.43E-23 | 0.2649 | 0.0266 | 0.7298 | 99.17357357 |  | 0.1965 | 0.0044 | 0.0034 | 0.633 |
| rs9275390 | C | T | 1.00E-200 | 1.0547 | 0.0281 | 0.4364 | 1408.771547 |  | 0.00419904 | 0.0109 | 0.0038 | 0.2515 |
| rs9468618 | T | C | 1.09E-14 | -0.4185 | 0.0542 | 0.05602 | 59.61940959 |  | 0.7792 | -0.0016 | 0.0058 | 0.0882 |

SNP: single nucleotide polymorphism; A1: effect allele; A2: other allele; SE: standard error; EAF: effect allele frequency.

**Supplementary Table 8.** Detailed Description of 4 SNPs Associated with Frailty in Multiple Sclerosis.

| SNP ID | A1 | A2 | Exposure (Hypothyroidism) | | | | |  | Outcome (Frailty) | | | |
| --- | --- | --- | --- | --- | --- | --- | --- | --- | --- | --- | --- | --- |
|  |  |  | P value | Beta | SE | EAF | F-statistic |  | P value | Beta | SE | EAF |
| rs141298848 | T | C | 2.67E-08 | 0.8046 | 0.1447 | 0.02965 | 30.91854252 |  | 0.8231 | -0.0029 | 0.013 | 0.0187 |
| rs55782725 | A | G | 3.62E-13 | 0.9198 | 0.1265 | 0.03668 | 52.8690831 |  | 0.437 | -0.0053 | 0.0068 | 0.0624 |
| rs9264277 | C | T | 1.67E-18 | -0.4545 | 0.0518 | 0.73 | 76.98467394 |  | 0.1965 | 0.0044 | 0.0034 | 0.633 |
| rs9271069 | G | A | 1.54E-58 | -1.1429 | 0.0708 | 0.8612 | 260.5829337 |  | 4.99E-07 | 0.0231 | 0.0046 | 0.8492 |

SNP: single nucleotide polymorphism; A1: effect allele; A2: other allele; SE: standard error; EAF: effect allele frequency.

**Supplementary Table 9.** Detailed Description of 38 SNPs Associated with Frailty in Overall Autoimmune Disease.

| SNP ID | A1 | A2 | Exposure ( Overall Autoimmune Disease ) | | | | |  | Outcome (Frailty) | | | |  |
| --- | --- | --- | --- | --- | --- | --- | --- | --- | --- | --- | --- | --- | --- |
|  |  |  | P value | Beta | SE | EAF | F-statistic |  | P value | Beta | SE | EAF |  |
| rs10081055 | G | T | 9.48E-09 | -0.0574 | 0.01 | 0.2773 | 32.94729882 |  | 0.2639 | 0.004 | 0.0035 | 0.3264 |  |
| rs10088596 | C | T | 1.91E-09 | -0.0563 | 0.0094 | 0.6609 | 35.87212569 |  | 0.753999 | 0.0012 | 0.0037 | 0.7187 |  |
| rs10116520 | G | A | 3.42E-14 | 0.0685 | 0.009 | 0.4011 | 57.92848281 |  | 0.00463298 | 0.0096 | 0.0034 | 0.3855 |  |
| rs10844737 | C | A | 2.14E-11 | 0.0674 | 0.0101 | 0.262 | 44.53208974 |  | 0.2992 | 0.0038 | 0.0037 | 0.2753 |  |
| rs10905669 | T | C | 3.23E-16 | 0.0802 | 0.0098 | 0.2854 | 66.97189925 |  | 0.2503 | 0.0045 | 0.0039 | 0.2322 |  |
| rs11571297 | C | T | 1.06E-33 | -0.1109 | 0.0092 | 0.3711 | 145.3059732 |  | 0.0883995 | -0.0056 | 0.0033 | 0.486 |  |
| rs12135754 | C | A | 5.20E-09 | -0.084 | 0.0144 | 0.1072 | 34.02746673 |  | 0.5927 | 0.003 | 0.0057 | 0.0923 |  |
| rs12575636 | G | T | 3.23E-10 | 0.0724 | 0.0115 | 0.1799 | 39.63487399 |  | 0.5632 | 0.0024 | 0.0042 | 0.189 |  |
| rs13105678 | A | C | 1.65E-08 | 0.0514 | 0.0091 | 0.5798 | 31.90358471 |  | 0.0878092 | 0.0058 | 0.0034 | 0.598 |  |
| rs13220783 | T | G | 3.86E-09 | 0.0537 | 0.0091 | 0.3815 | 34.82264992 |  | 0.3547 | 0.0031 | 0.0034 | 0.3975 |  |
| rs139805598 | T | C | 1.00E-08 | -0.1469 | 0.0256 | 0.03172 | 32.92757071 |  | 0.0302099 | 0.036 | 0.0166 | 0.0103 |  |
| rs1683253 | C | T | 1.77E-09 | 0.0537 | 0.0089 | 0.5563 | 36.40529781 |  | 0.6605 | 0.0014 | 0.0033 | 0.4804 |  |
| rs1915930 | T | G | 3.50E-15 | 0.0719 | 0.0091 | 0.618 | 62.42679319 |  | 0.741 | 0.0011 | 0.0033 | 0.5481 |  |
| rs1990760 | T | C | 6.41E-14 | 0.0674 | 0.009 | 0.5849 | 56.08294413 |  | 0.01438 | -0.0083 | 0.0034 | 0.6087 |  |
| rs2017445 | A | G | 6.94E-11 | 0.066 | 0.0101 | 0.2913 | 42.70130557 |  | 0.7086 | -0.0013 | 0.0035 | 0.3425 |  |
| rs2281388 | A | G | 3.34E-14 | -0.2272 | 0.03 | 0.02305 | 57.35485349 |  | 0.00310099 | -0.0334 | 0.0113 | 0.0216 |  |
| rs2293158 | C | T | 9.75E-09 | -0.0598 | 0.0104 | 0.2369 | 33.06219777 |  | 0.4269 | -0.0029 | 0.0036 | 0.3033 |  |
| rs244686 | C | T | 1.51E-09 | -0.0616 | 0.0102 | 0.7486 | 36.47179271 |  | 0.2332 | -0.006 | 0.005 | 0.8748 |  |
| rs2516466 | G | T | 1.66E-15 | 0.0745 | 0.0094 | 0.4416 | 62.81348194 |  | 0.1825 | 0.0044 | 0.0033 | 0.552 |  |
| rs2747442 | G | A | 2.12E-12 | -0.0764 | 0.0109 | 0.2115 | 49.12807545 |  | 0.770699 | -0.0011 | 0.0036 | 0.2976 |  |
| rs2847258 | T | C | 2.02E-08 | -0.0498 | 0.0089 | 0.4783 | 31.30939692 |  | 0.4617 | -0.0025 | 0.0033 | 0.4248 |  |
| rs3104415 | C | A | 4.00E-98 | 0.2257 | 0.0107 | 0.4436 | 444.9299008 |  | 4.17E-12 | 0.0241 | 0.0035 | 0.3406 |  |
| rs310772 | A | G | 1.05E-08 | -0.0603 | 0.0105 | 0.7723 | 32.98010669 |  | 0.0425305 | -0.0077 | 0.0038 | 0.7504 |  |
| rs35789143 | A | G | 3.94E-10 | 0.0747 | 0.0119 | 0.1646 | 39.40427224 |  | 0.2288 | 0.006 | 0.005 | 0.1288 |  |
| rs3842753 | G | T | 2.45E-12 | 0.0771 | 0.011 | 0.7913 | 49.12690629 |  | 0.7656 | 0.0011 | 0.0037 | 0.7103 |  |
| rs4073285 | T | C | 3.90E-10 | -0.0577 | 0.0092 | 0.3626 | 39.33435216 |  | 0.1509 | -0.005 | 0.0035 | 0.3335 |  |
| rs4151670 | T | C | 7.21E-27 | -0.2673 | 0.0249 | 0.03403 | 115.2378782 |  | 0.3114 | -0.0113 | 0.0112 | 0.0228 |  |
| rs4853458 | G | A | 2.01E-26 | -0.1117 | 0.0105 | 0.7688 | 113.1680358 |  | 0.1334 | -0.006 | 0.004 | 0.7774 |  |
| rs6679677 | A | C | 3.84E-149 | 0.3269 | 0.0126 | 0.1472 | 673.1080445 |  | 0.001736 | 0.0171 | 0.0055 | 0.1018 |  |
| rs71508903 | T | C | 9.57E-10 | 0.0699 | 0.0114 | 0.1853 | 37.59591672 |  | 0.6417 | 0.002 | 0.0042 | 0.1928 |  |
| rs7205474 | T | C | 3.51E-08 | -0.0641 | 0.0116 | 0.1761 | 30.53487248 |  | 0.4116 | 0.0038 | 0.0046 | 0.1543 |  |
| rs76169968 | A | G | 1.99E-10 | -0.095 | 0.0149 | 0.09841 | 40.65095041 |  | 0.4507 | -0.0045 | 0.006 | 0.0835 |  |
| rs7823488 | T | C | 2.62E-08 | 0.1192 | 0.0214 | 0.04458 | 31.02565752 |  | 0.0169099 | -0.0234 | 0.0098 | 0.0294 |  |
| rs7850258 | G | A | 6.49E-43 | 0.1278 | 0.0093 | 0.6536 | 188.8390646 |  | 0.7563 | -0.0011 | 0.0035 | 0.6684 |  |
| rs9273363 | A | C | 1.00E-200 | 0.3697 | 0.0105 | 0.2794 | 1239.699235 |  | 4.98E-05 | 0.0146 | 0.0036 | 0.303 |  |
| rs9497965 | T | C | 3.24E-09 | 0.0555 | 0.0094 | 0.337 | 34.85991221 |  | 0.4345 | 0.0026 | 0.0034 | 0.4086 |  |
| rs9687206 | G | A | 9.09E-25 | 0.0913 | 0.0089 | 0.4672 | 105.2343619 |  | 0.2038 | -0.0043 | 0.0034 | 0.4272 |  |
| rs9863204 | G | A | 2.31E-08 | 0.0522 | 0.0093 | 0.3387 | 31.50439463 |  | 0.9882 | -1.00E-04 | 0.0036 | 0.3085 |  |

SNP: single nucleotide polymorphism; A1: effect allele; A2: other allele; SE: standard error; EAF: effect allele frequency.
